# Odor Mixtures in Identification Testing Using Sniffin’ Sticks: The SSomix Test

**DOI:** 10.1038/s41598-020-65028-7

**Published:** 2020-05-18

**Authors:** David Tianxiang Liu, Gerold Besser, Miriam Lang, Gunjan Sharma, Eleonore Pablik, Bertold Renner, Christian Albert Mueller

**Affiliations:** 1 Department of Otorhinolaryngology, Head and Neck Surgery, Medical University of Vienna, Vienna, Austria; 2 Section for Medical Statistics, CeMSIIS, Medical University of Vienna, Vienna, Austria; 3 Institute of Experimental and Clinical Pharmacology and Toxicology, Friedrich-Alexander-Universität Erlangen-Nürnberg, Erlangen, Germany; 4 Institute of Clinical Pharmacology, Medizinische Fakultät Carl Gustav Carus, Technische Universität Dresden, Dresden, Germany

**Keywords:** Olfactory system, Diagnosis

## Abstract

Clinical tests assessing olfactory performance have become indispensable for diagnosing olfactory dysfunction. As time and personnel resources are limited, it would be advantageous to have shorter protocols focusing on singular aspects of olfactory performance, such as odor identification. However, such a unidimensional approach is often inconclusive and needs further tests (and tools). Hence, new testing methods with high levels of sensitivity, specificity, and reproducibility are required for clinical practice. Here, we developed a Sniffin’ Sticks odor mixture identification test method (SSomix), with emphasis on resource efficiency and simplicity of administration. SSomix consists of mixtures of two and three odors applied onto a piece of paper using 11 out of 16 items from the original Sniffin’ Sticks identification test kit. A total of 66 healthy subjects and 22 patients with olfactory dysfunction were included in the study. SSomix showed good to excellent test-retest reliability and validity. The area under the receiver operating characteristics curves indicated good diagnostic accuracy in identifying patients with reduced and severely impaired olfactory function. SSomix was a suitable downsizing of the original kit, especially regarding resource efficiency.

## Introduction

The sense of smell is critical for our perception of the environment, and olfactory dysfunction represents a major loss of ambient information. The causes of such a condition are diverse, including head traumas, neurodegenerative disorders, and upper airway infections, or else it could be idiopathic^1^. Olfactory performance is known to decrease in the elderly and has a profound effect on the safety and quality of life of affected individuals^2,3^. Indeed, testing has become an integral part of diagnosing olfactory dysfunction. Short screening protocols that focus on one subsidiary dimension of olfactory testing, such as odor identification, are favoured in clinical routine. However, reproducibility issues in longitudinal settings, due to the small number of items used for these tests (16items) have led to the development of extended versions, by adding 16 additional odors to the same protocol^4,5^. Faced with the aforementioned initial obstacles of short tests in longitudinal follow-up testing, utilizing a pre-existing tool we developed a new test method that reduced the number of items used but simultaneously increased mental workload during testing. The tool used, termed the Sniffin’ Sticks odor mixture identification test method (SSomix), is based on the identification of single odors in binary and trinary mixtures. We hypothesized that this increased workload could lead to high test-retest reliability and diagnostic accuracy compared to the benchmark. Moreover, since odor mixtures represent daily life situations more accurately compared to single odorants, increased mental workload during olfactory testing might also be useful to reveal disease-specific differences in neurodegenerative and mood disorders^6^.

In order to increase accuracy in olfactory tests, it is recommended to determine olfactory performance in a combined approach by testing the three olfactory dimensions of threshold, discrimination, and identification (TDI)^7,8^. One method for olfactory testing is represented by Sniffin’ Sticks, which are based on impregnated felt tip pens^7,8^. Extended versions of Sniffin’ Sticks adding more odored pens to the standard version (more than 16) have also shown higher accuracy and test-retest reliability^5,9^. However, these extended versions are too time consuming to be practical in a clinical setting.

Screening tests are usually unidimensional and based on odor identification. Previous studies have suggested that odor thresholds pertain more to peripheral sensory capacity whereas discrimination and identification are more related to higher level cognitive function^10,11^. Due to these findings but possibly also to easy availability and time efficiency, many studies on neurodegenerative diseases have focused solely on olfactory identification ability^12,13^. It has been previously postulated that increasing the mental workload by adding complexity to identification testing might further reveal disease-specific differences in olfactory performance between Parkinson’s and Alzheimer’s diseases (PD and AD, respectively)^14^. Furthermore, introducing a test that is based on the presentation of odors with different values of hedonic tone may also be important for future studies of patients with mood disorders (such as depression), in which pleasure-loss in commonly pleasant experiences (anhedonia) occurs as a major symptom^6^. Current knowledge and methodology in these field are sparse.

With regard to the test-retest reliability of olfactory tests, results have been traditionally reported on the sole basis of correlation coefficients (mainly Pearson’s *r*^8,15–20^). Since this coefficient only refers to the degree of association, it lacks the ability to discriminate between the extent to which two measurements are identical (agreement)^21,22^. This shortcoming highlights the need to meet the criteria of reproducibility during the development of new olfactory tests^21–24^.

In the present work, we refined the German version of the 16-item Sniffin’ Sticks identification test and evaluated a new screening test protocol to assess olfactory performance based on SSomix. It utilizes 13 mixtures of 11 odors (grouped into two or three) selected from the 16-item identification test, based on odor identification combined with a procedure using painted lines on a piece of paper (“Odor-Lines-On-Paper”)^25,26^. The aim was to evaluate reproducibility and validate the SSomix test method in comparison to a benchmark test for olfactory performance.

## Results

### SSomix distinguishes between self-reported normal and impaired olfactory function

To determine whether our abridged protocol (11 odors) would be as efficient as the original Sniffin’ Sticks test (TDI) for assessing self-reported olfactory dysfunction, both methods were applied to a cohort of 22 patients and 66 healthy volunteers. Odors were marked on standard print paper and participants were asked to identify the two or three odors used during two separate visits. Scores for TDI and SSomix tests were calculated for the initial visit of subjects with self-reported olfactory dysfunction and control participants. Participant answers were recorded by the authors and the results analysed using the Welch t-test.

Descriptive statistics of the SSomix test scores for the first visits of healthy volunteers are listed in Supplementary Table 1. The results showed that control participants had Sniffin’ Sticks test scores (*M* ± *SD*) of 33.3 ± 4.5 and SSomix test scores (*M* ± *SD*) of 18.5 ± 3.6 for the first visit^27,28^. The results additionally showed that the respective TDI and SSomix test scores (*M* ± *SD*) for the patients were lower, at 17.5 ± 6.3 and 7.8 ± 3.2. TDI and SSomix test scores were compared between the control and patient groups and an unpaired t-test with Welch’s correction showed these differences to be significant (for TDI, *t*(28.4) = 10.9, *p* < 0.001; for SSomix, *t*(39.8) = 13.2, *p* < 0.001).

Hence, the abridged SSomix test appeared to be efficient at distinguishing olfactory performance between subjects with self-reported normal sense of smell and olfactory dysfunction. SSomix was then assessed for reproducibility by determining to what extent the scores obtained during subsequent visits could be replicated.

### SSomix shows good to excellent test-retest reliability

The test-retest reliability and agreement of the SSomix test method was assessed using the intraclass correlation coefficient (ICC)^29^ and the Bland-Altman statistical methodology^30^.

**Figure 1:**
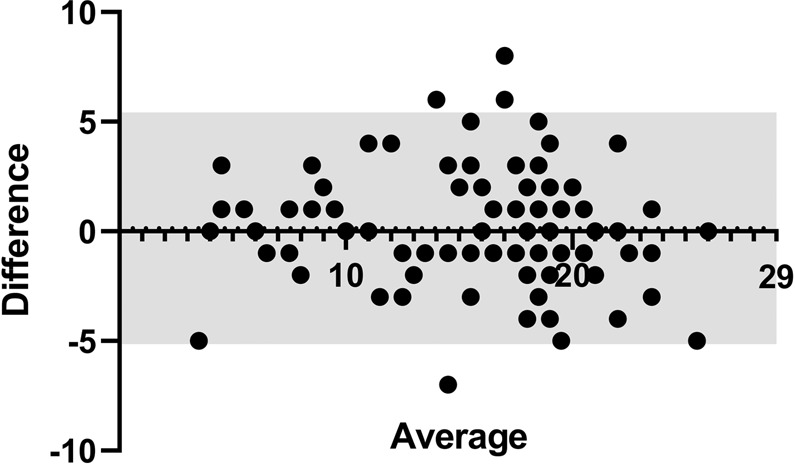
Bland Altman plot of the SSomix scores of all subjects (n = 88). Differences between SSomix scores from the first and second visits were plotted against the average scores of the two visits, 95% limits of agreement are indicated within the grey area (from -5.15 to 5.42), bias (mean difference) is indicated by the horizontal dotted line (0.14). Abbreviations: Difference = Differences between SSomix scores from the first and second visit, Average = Average SSomix scores from the first and second visits.

The results showed good to excellent test-retest reliability (ICC = 0.89, 95% confidence interval from 0.83 to 0.92, (*F*(87,87) = 17, *p* < 0.001); Fig. 3A) for the SSomix test. We also calculated the bias which is the mean difference in the SSomix test scores between first and second visits as well as the 95% limits of agreement. The bias of nearly zero (0.14) and 95% limits of agreement from −5.15 to 5.42 indicated neither a systemic nor a proportional error (Fig. 1).

Since the SSomix test seemed to be reliable and showed agreement, it would also be interesting to determine whether there might be an association with more widely used and well-established tests for olfactory performance.

### SSomix correlates significantly with the Sniffin’ Sticks test

**Figure 2:**
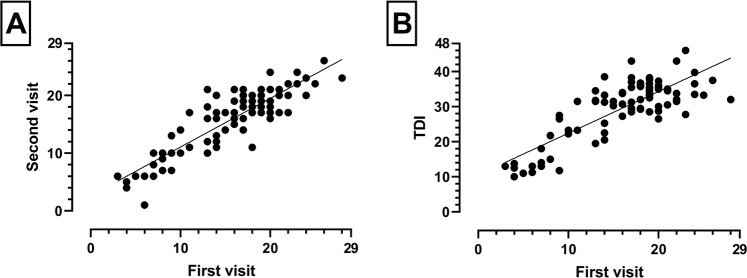
(**A**) Scatter plot of SSomix scores between the first and second visits, straight line showing line of regression (Intraclass correlation coefficient = 0.89, p < 0.001). (**B**) Scatter plot between SSomix and TDI scores from the first visit (Spearman correlation = 0.69, p < 0.001).

To assess the validity of the SSomix test, we correlated the results obtained during the first and second visits with the results of the Sniffin’ Sticks test as well as the individual subtests. The SSomix test results from both visits showed a moderate to strong correlation with the TDI (for the first visit, *r*(88) = 0.69, *p* < 0.001, Fig. 2; for the second visit, *r*(88) = 0.65, both *p* < 0.01) as well as the subtests (for the first visit: for T, *r*(88) = 0.49; for D, *r*(88) = 0.65; for I, *r*(88) = 0.67; for TD, *r*(88) = 0.63; for TI, *r*(88) = 0.65, for DI, *r*(88) = 0.72; all *p* < 0.001) with only minor fluctuations between visits.

Since the SSomix test also appeared to show a strong correlation with a well-established test for olfactory performance, we were interested to determine the accuracy of the SSomix test in identifying patients with olfactory dysfunction compared to the benchmark.

### SSomix shows good diagnostic accuracy

The Sniffin’ Sticks test allows the classification of patients into three different groups according to the number of points achieved by comparison with normative data^27,28,31^: (i) normal olfactory function, normosmic, (ii) reduced olfactory function, hyposmic, and (iii) severe olfactory dysfunction, anosmic. Subjects classified as anosmic (TDI ≤ 16; n = 12) had an SSomix test score of *M* ± *SD* (range) of 5.7 ± 1.8 (3-9). We then grouped the hyposmic (n = 25) and anosmic (n = 12) cohorts together and defined a TDI of less than 30.75 (as previously described^28^) so as to distinguish between normosmic (n = 51) and hyposmic/anosmic (n = 37) subjects for further analysis. Subjects in the normosmic group had an SSomix test score *M* ± *SD* (range) of 18.7 ± 3.7 (11-28), which was significantly higher compared to the hyposmic/anosmic group 11.8 ± 5.8 (3-23; *t*(56.1) = 6.4, *p* < 0.001).

**Figure 3:**
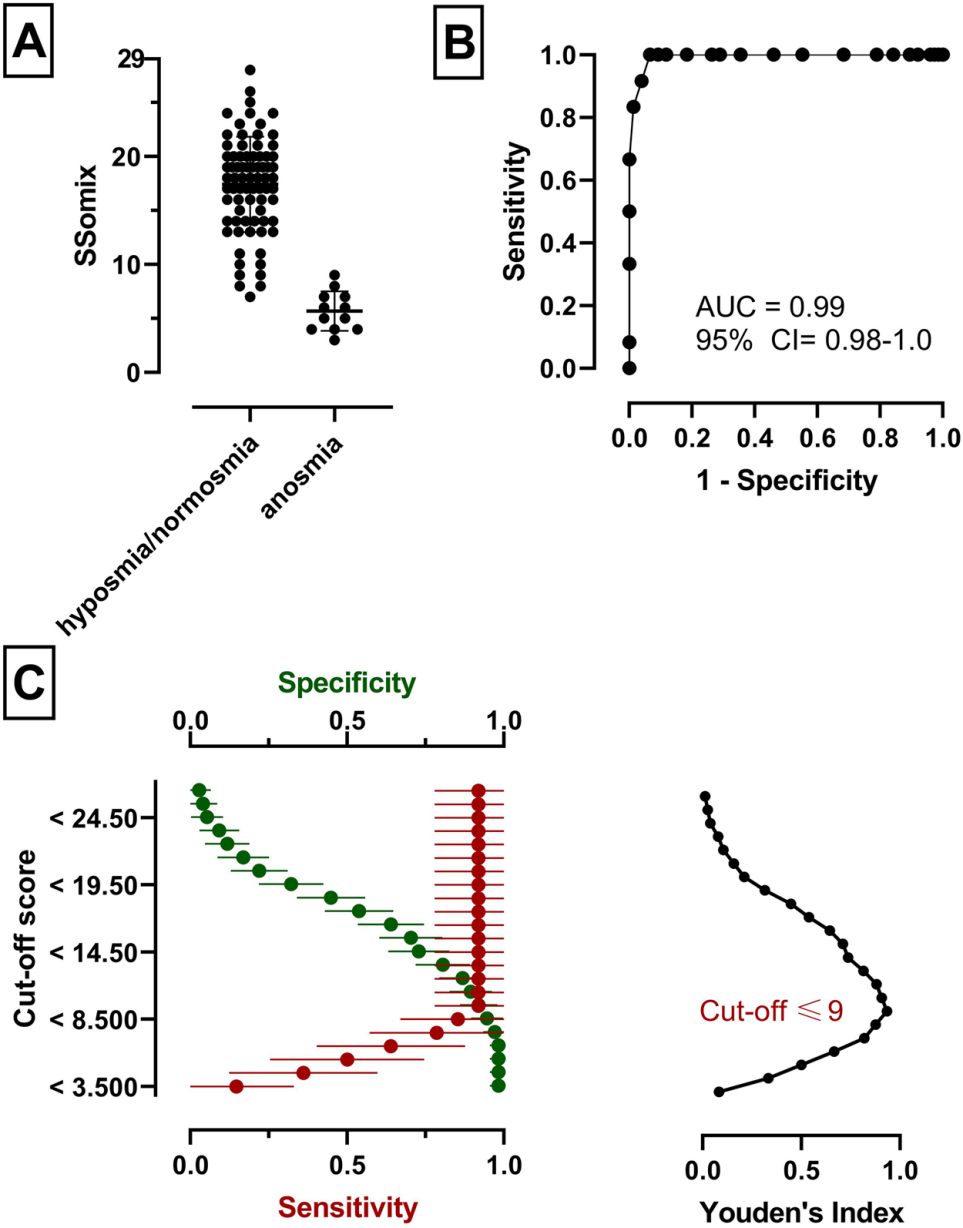
Diagnostic accuracy of SSomix to distinguish between ansomia from hyposmia/normosmia. (**A**) Comparison of SSomix scores from subjects divided into hyposmia/normosia and anosmia, middle line showing mean value. (**B**) Area under the receiver operating curve (AUC) to distinguish between anosmia from hyposmia/normosmia. (**C**) Sensitivity and specificity for different cut-off scores (respective percentage and 95% confidence interval). The optimal cut-off score is indicated by the red plot (Youden’s Index).

**Figure 4:**
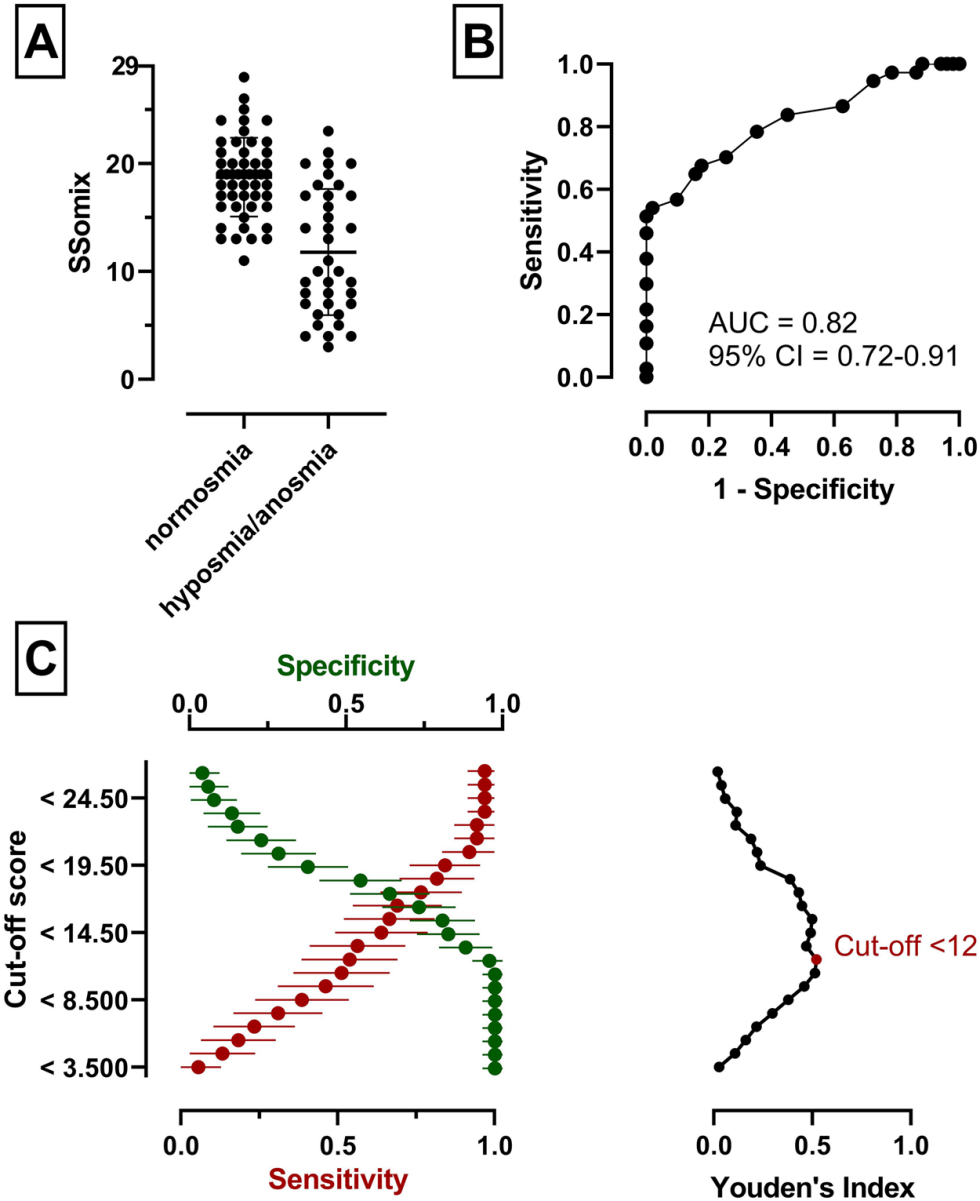
Diagnostic accuracy of SSomix to distinguish between normosmia from hyposmia/anosmia. (**A**) Comparison of SSomix scores from subjects divided into normosia and hyposmia/anosmia, middle line showing mean value. (**B**) Area under the receiver operating curve (AUC) to distinguish between normosmia from hyposmia/anosmia. (**C**) Sensitivity and specificity for different cut-off scores (respective percentage and 95% confidence interval). The optimal cut-off score is indicated by the red plot (Youden’s Index).

We then calculated the diagnostic accuracy of the SSomix test to discriminate between the anosmic and hyposmic/normosmic groups and the normosmic and hyposmic/anosmic groups, by calculating the area under the receiver operating curve (AUC). The AUC of 0.99 (95% confidence interval from 0.98 to 1.0; Fig. 3) reflected a test with nearly no false positive or negative results to discriminate between anosmia and hyposmia/normosmia. The optimal cut-off score (for anosmia) was determined as ≤9.0 (sensitivity 1.0, specificity 0.93) by using the Youden Index, which maximizes the ability of a test to differentiate giving equal importance to sensitivity and specificity^32^. The obtained AUC of 0.82 (95% confidence interval from 0.72 to 0.91; Fig. 4) reflected good diagnostic accuracy in identifying subjects with olfactory dysfunction. Using this method, the optimal cut-off score (for dysosmia) was determined as <12 (sensitivity 0.54, specificity 0.98).

## Discussion

Tests for olfactory performance have become indispensable in the diagnosis of olfactory dysfunction. Comprehensive tests for olfactory performance such as the Sniffin’ Sticks test battery, including all three components of threshold, discrimination, and identification testing, can take up to one hour, requiring additional personnel resources. For this reason, shorter screening protocols such as the Sniffin’ Sticks identification test^33–42^ were frequently administered in clinical practice. These screening tests became increasingly popular and extensively used worldwide, but were often less accurate and inconclusive^17,18^. Therefore, shorter SSomix protocols were considered, based on the presentation of 13 different odor mixtures in identification testing, utilizing only the commonly available 16-item Sniffin’ Sticks identification test. Here, we show that SSomix was efficient in distinguishing between self-reported normal olfactory function and dysfunction, which is a basic test requirement for olfactory performance^43^. In addition, the reproducibility of SSomix was also verified as test-retest results in patients and controls were essentially identical and showed a good to excellent test-retest reliability. Moreover, SSomix appeared to be a valid test for olfactory performance since it correlated significantly with the established Sniffin’ Sticks test battery (TDI), the gold standard in the field. Finally, SSomix was demonstrated to have good diagnostic accuracy in our tested cohort.

In reference to the efficiency of SSomix for distinguishing between self-reported normal olfactory function and dysfunction, although our method eliminated various steps from the gold standard test protocol (from three to one), within our cohort it was still possible to determine with confidence the difference between normal and dysfunctional olfactory performance. Previous studies attempted to curtail the length of the identification test by removing elements^17,18^, however, diagnostic accuracy and reproducibility in terms of test-retest agreement remained an issue. The present method seemed to have overcome these problems. Since SSomix was based only on the 16-item Sniffin’ Sticks identification test and therefore easy to administer, it could be used in addition to shorter protocols (e.g. identification screening tests) and would require very few material and financial resources. This should enable physicians to provide a more accurate statement regarding olfactory function in cases of subjective loss of smell and unclear test results. Accordingly, for screening of anosmic patients, the SSomix test cut-off score of nine and lower correctly identified all anosmic patients.

The development of a new outcome measure is a continuous process by which information is collected and analysed to demonstrate scientific reproducibility and validity^44^. As a result, the reproducibility in terms of test-retest reliability and agreement plays a significant role in the evaluation of new procedures^22^. While the latter describes the extent to which two measurements are identical^30^, test-retest reliability usually refers to the ability of a measurement to generate constant and similar results^22,24,29,45^. Concerning the reproducibility of odor identification tests, these quality estimates have been traditionally examined on the basis of the correlation coefficients Pearson’s *r*^8,15–20^, Spearman’s rho^46,47^, or Lin’s concordance^48^. One previous investigation reported Pearson’s *r* and agreement measures^5^. Furthermore, simple linear regression analysis was also used to conclude test-retest reliability^49^. Here, we used the intraclass correlation coefficient and Bland-Altman statistical method to demonstrate test-retest reliability and agreement, which was commensurate with previously advocated methods relating to reproducibility^21,22,50^. It is worth noting that the inter-individual olfactory function of included participants can also have a profound effect on correlation coefficients (e.g. Pearson’s *r*) in test-retest studies. In other words, studies including subjects with a wide range of olfactory function (normosmic and anosmic patients) tend to show higher test-retest correlation coefficients compared to studies including subjects with a smaller range of olfactory function (e.g. solely including normosmic patients)^51^. Therefore, in terms of correlation coefficients alone, test-retest reliabilities should be interpreted and compared across studies with caution and rather seen as part of the test in a given sample and under given experimental circumstances^22^.

As indicated above, a further important aspect to our work was the reproducibility of SSomix. The correlation coefficient of 0.89 and the Bland-Altman plot showing neither a systemic nor a proportional error demonstrated high test-retest reliability and agreement between both measurements. Higher test-retest reliabilities in terms of correlation coefficients have been shown in extended versions of the Sniffin’ Sticks subtests, which was suggested to be the result of a larger variability in the test scores (32 instead of 16 points^5,19^). The maximum attainable score of 29 in the SSomix test method may also be a reason for the good to excellent test-retest reliability. Odor threshold, which is the ability to recognize a minimum quantity of a single odor molecule, is usually tested using n-butanol^7,27^. An increased number of activated olfactory receptors using more varied molecules in threshold testing was suggested to be the reason for higher correlation coefficients between two measurements of the same cohort^52^. We assumed that the combination of hedonically matched, non-overlapping, and familiar odors might also lead to increased receptor activation, thereby resulting in the good to excellent test-retest reliability and agreement observed in our study^53^.

The SSomix test method was developed for identification of single components in binary/trinary mixtures. Therefore, we assumed that the correlation coefficient between SSomix and the combination of the identification and discrimination subtests of the Sniffin’ Sticks test should be highest, which was eventually demonstrated by our results. Interestingly, the threshold subtest also revealed a moderate correlation with SSomix, underlining the close interdependence of odor identification, discrimination, and threshold. A study was previously undertaken^54^ that was aimed at addressing how different subtests of the Sniffin’ Sticks test contribute to the assessment of olfactory function in patients with PD and severe olfactory dysfunction compared to healthy controls. It revealed that the sole determination of threshold, discrimination, or identification cannot replace the more comprehensive diagnostic approach of determining all three qualities. This is because odor threshold, in contrast to discrimination and identification, showed distinctive characteristics in the healthy control group. On the other hand, in patients with PD and severe olfactory impairment, individual qualities of threshold, discrimination, and identification showed higher sensitivity and specificity in the correct diagnosis of olfactory dysfunction. This observation was suggested to be the result of an increase in similarity within all three qualities with decreasing olfactory function in patients with PD^54^. Our results support these findings, since the sensitivity and specificity of SSomix also increased with decreasing olfactory function.

The decision to include binary and trinary mixtures was influenced by the consideration of olfactory dysfunctions associated with neurologic disorders such as PD and AD^12,13^. While it is well known that both PD and AD are associated with disorders of the sense of smell (especially regarding odor discrimination abilities), subtle differences in tasks involving odor identification, threshold, and recognition have suggested that AD patients are more affected in higher level olfactory abilities involving cognitive processes compared to PD patients that are more affected in lower level olfactory tasks^14^. Considering this theory, it is tempting to speculate on a difference in the number of correctly identified odors in binary and trinary mixtures (which subsequently increases mental workload) as compared to regular “one-odor” identification tasks between AD and PD patients with smell disorders. These potential patterns might provide further evidence for differences in olfactory abilities between AD and PD and should therefore be investigated in future studies.

The accuracy of SSomix was found to be most pronounced in patients with severe olfactory dysfunction. As mentioned above, accelerating clinical work in these patients by utilizing shorter tests usually comes at the expense of diagnostic accuracy and test-retest reliability^17,18,47^. Patients diagnosed with ansomia using the TDI test yielded a maximum SSomix score of nine out of 29 points compared to a maximum score of eight out of 12 points using the 12-item identification screening test^17^. Extended versions of established tests may yield more detailed information for assessment of olfactory performance, as smaller differences can be detected by allowing a larger variability in data gathered^5,19^. It follows that the 29-point scoring could have positively affected the diagnostic accuracy of SSomix.

In summary, SSomix seemed to be a reliable, accurate, and short method requiring only the 16-item Sniffin’ Sticks identification test to assess olfactory function in a novel approach to odor identification and could be used as a screening tool or in addition to shorter protocols. Similar to the SSomix test method, “Odor-Lines-On-Paper” have already been proposed for self-administered testing of all Sniffin’ Sticks subtests^25,26^. Based on this method, clinicians may benefit from an additional test method using available tools thereby providing the possibility of self-administration.

## Methods

### Ethics statement

This study was approved by the Ethics Committee of the Medical University of Vienna (EK-Nr.: 1165/2018) and conducted according to the Declaration of Helsinki on biomedical research involving human subjects at the Department of Otorhinolaryngology, Medical University of Vienna (between March 2018 and June 2019). Written informed consent was obtained from all subjects prior to the participation.

### Subjects

91 subjects (52 females, 39 males; mean age ± *SD* (range) = 38.1 ± 18.3 years; 18-84 years) consisting of 68 subjects with self-reported normal sense of smell and no history of prior olfactory testing and 23 subjects with self-reported olfactory dysfunction were initially recruited for this study. A complete Ear, Nose, and Throat examination including the subject’s history and nasal endoscopy was performed in all participants. Subjects with current or past conditions which might affect olfactory function were excluded. Exclusion criteria for subjects with self-reported normal sense of smell were: (i) neurodegenerative diseases, (ii) acute or chronic rhinosinusitis^55^, (iii) smoker (>5 cigarettes/day), and (iv) a history of head trauma. The exclusion criteria were not applied to subjects with self-reported olfactory dysfunction. Two subjects with self-reported normal sense of smell and one subject with self-reported olfactory dysfunction declined further contact after the first visit resulting in a total of 88 subjects (51 females, 37 males; mean age ± *SD* (range) = 37.9 ± 18.2; 18-84 years), consisting of 66 healthy subjects (40 females, 26 males; mean age ± *SD* (range) = 30.2 ± 11.5; 18-77 years) and 22 subjects with anamnestic olfactory dysfunction (11 females, 11 males; mean age ± *SD* (range) = 61.3/14.1; 30-84 years) who were included in this study and tested twice on two different days.

### Study design

The SSomix test was performed twice with at least one day between tests (*M* ± *SD* = 34.7 ± 50.6 days). All subjects were tested using the Sniffin’ Sticks test (TDI)^7,8^ on the day of the first visit after the SSomix test in order to allow a classification according to published TDI cut-off scores and to validate the results with an established test for olfactory performance. All tests were performed in a well-ventilated room and feedback was not given to subjects before the end of the second visit.

### Development of SSomix

The development of this new method focused on the following points: (i) wide availability and pre-existing tools, (ii) simple procedure for self-administration, and (iii) suitability of a new test for olfactory performance, including high test-retest reliability, agreement, validity, and diagnostic accuracy.

We chose the 16-item Sniffin’ Sticks identification test to serve as the technical basis, since these 16 items are represented in the most widely used olfactory assessment tool in German-speaking countries and have been further adapted and validated for various other countries^33–42^. Regarding the procedure, we agreed on using the “Odor-Lines-On-Paper” method which has been validated for odor identification, discrimination, and threshold testing in previous studies of our working group^25,26^ and has the potential for self-administration.

It has been shown that odor mixtures present the risk of antagonistic interactions between each component at olfactory receptor binding sites, which might result in mixtures not smelling like individual components (“configural”)^56^. To ensure that single components of presented odor mixtures remain distinguishable (heterogenous^6,57^), the authors (D.T.L, G.B., and C.A.M) assessed different mixtures based on two, three, and four odors in a preliminary experiment (Table 1). In view of the human capacity to identify up to four different components in odor mixtures, we chose to include the same maximum number of individual elements in the preliminary experiments^58^. Unpleasant odors (e.g. garlic, fish, and turpentine) from the 16-item Sniffin’ Sticks were excluded beforehand since it has been shown that mixtures including high concentrations of malodorants have the capacity to mask weaker (pleasant) components^59,60^.

**Table 1:** All mixtures that were assessed during the preliminary experiments. Abbreviation: Odors 1–4 = First to fourth odor of the odor mixture.

Odor 1	Odor 2	Odor 3	Odor 4
Orange	Lemon		
Banana	Orange		
Lemon	Clove		
Peppermint	Pineapple		
Apple	Anise		
Licorice	Banana		
Cinnamon	Coffee		
Pineapple	Rose		
Shoe leather	Licorice		
Banana	Cinnamon		
Apple	Coffee		
Clove	Peppermint		
Orange	Cinnamon		
Lemon	Coffee		
Cinnamon	Lemon		
Peppermint	Cinnamon	Coffee	
Rose	Clove	Licorice	
Pineapple	Licorice	Banana	
Banana	Orange	Cinnamon	
Shoe leather	Pineapple	Apple	
Peppermint	Pineapple	Banana	Cinnamon
Rose	Lemon	Licorice	Shoe leather
Orange	Coffee	Apple	Licorice

The final selection was made according to the following criteria: (i) odors fit hedonically together (no unpleasant odors) and (ii) balanced intensity/iso-intensity, in order to minimize one component masking the other components^56,59^. Mixtures were ranked using either fitting (2 points), maybe fitting (1 point), and not fitting (0 points) according to the above-mentioned criteria. Finally, eleven odors were selected based on ten combinations of two and three combinations of three different odors (according to preliminary results, ranked from highest to lowest). The eleven odors are: Anise, apple, banana, cinnamon, clove, coffee, lemon, orange, rose, peppermint, and pineapple. Mixtures were presented according to a given order, outlined in Table 2.

**Table 2:** Order in which the ten combinations of two odors and three combinations of three odors were presented. Abbreviation: Order = Order in which the mixtures were presented, Odors 1–3 = First to third odor of the odor mixture.

Order	Odor 1	Odor 2	Odor 3
1	Orange	Lemon	
2	Peppermint	Cinnamon	
3	Pineapple	Peppermint	
4	Coffee	Cinnamon	
5	Pineapple	Rose	
6	Banana	Cinnamon	
7	Apple	Coffee	
8	Clove	Peppermint	
9	Orange	Cinnamon	
10	Coffee	Lemon	
11	Orange	Coffee	Cinnamon
12	Banana	Pineapple	Rose
13	Lemon	Apple	Anise

**Figure 5:**
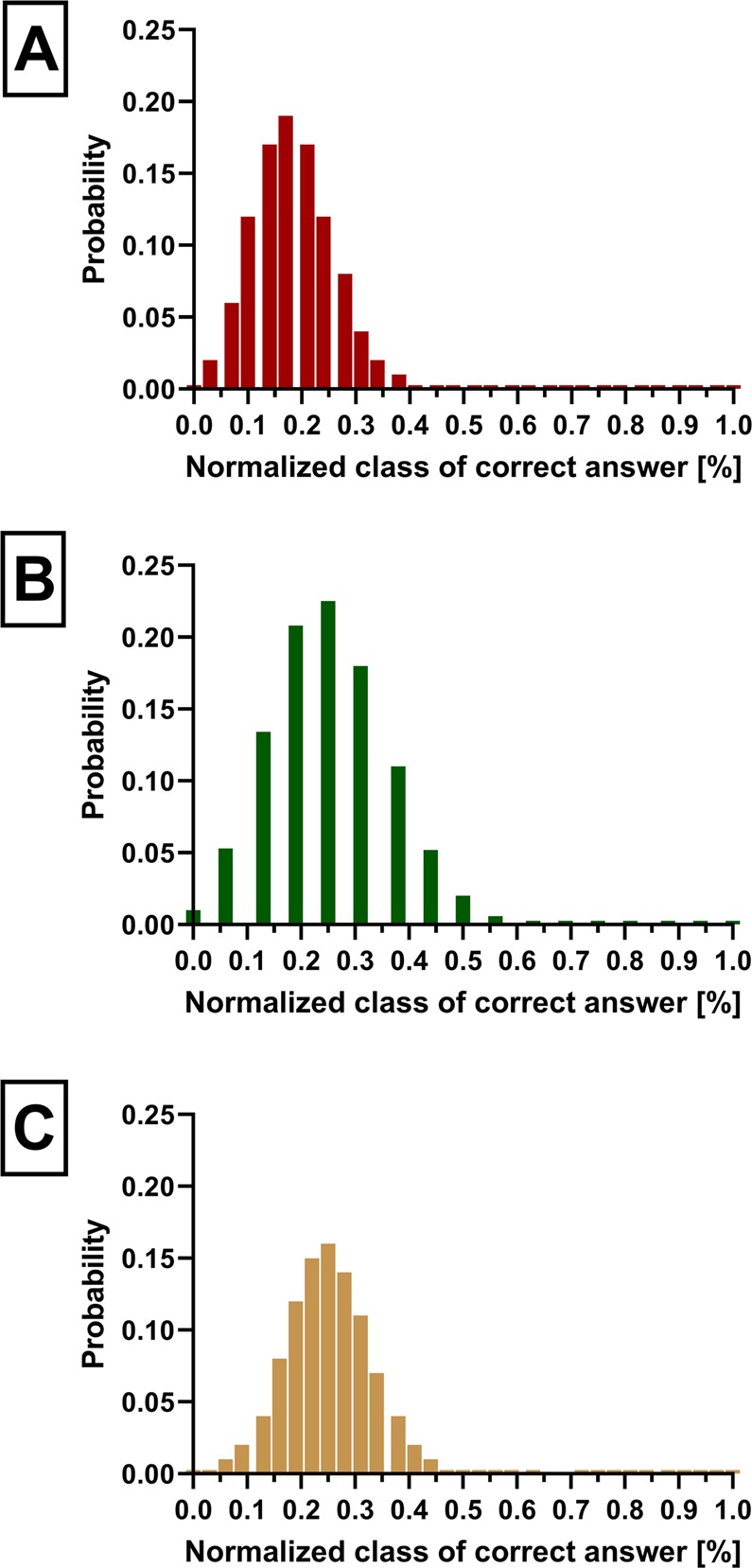
Theoretical chance-level responses (anosmics) calculated based on the binomial distribution. The x-axis represents the normalized number of correct answers from 0 to 100%. The y-axis represents the probability. (**A**) SSomix using an eleven, ten, and nine-alternative, forced-choice procedure (range 0–29), (**B**) the Sniffin’ Sticks 16-item identification test using a four-alternative, forced-choice procedure (range 0–16), and (**C**) the Sniffin’ Sticks 32-item identification test using a four-alternative, forced-choice procedure (range 0–32). Abbreviation: 16-item = 16-item Sniffin’ Sticks test, SSomix = SSomix test method, 32-item = 32-item Sniffin’ Sticks test.

The intention was to fit the time frame of less than fifteen minutes. Each correctly identified odor yielded one point, resulting in a score ranging from 0 to 29. Considering the theoretical chance levels of the four-alternative, forced-choice procedure used for the 16- and 32-item identification tests, we also aimed to reduce these levels for SSomix by increasing the maximum achievable number of points (from 16 to 29) and implementing more answer options. Since SSomix uses both binary and trinary mixtures of eleven different odors, theoretical chance levels vary between each response (eleven-, ten-, and nine-alternative, forced-choice procedure) and have been calculated based on the binomial probability distribution (Fig. 5).

### Testing protocol

**Figure 6:**
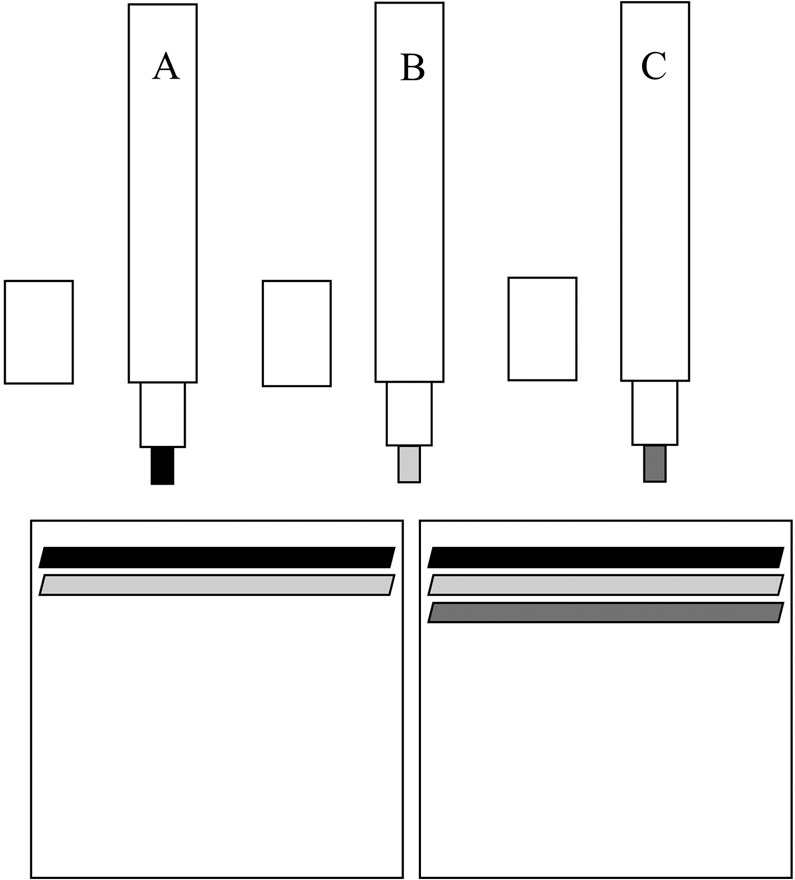
“Odor-Lines-On-Paper”: One piece of paper (6 × 6 cm) on which different identification Sniffin’ Sticks were applied horizontally over a length of 5 cm with a minimum intervening distance to prepare the odor mixtures which were then presented. Abbreviations: A, B, C = Three different identification Sniffin’ Sticks.

Subjects were given a list with eleven descriptors sorted in alphabetical order at the beginning of the test and the instruction to place the piece of paper in front of both nostrils^25,26^. The task was to identify each individual odor of the odor mixtures from the list of eleven descriptors. Mixtures were prepared by the investigator in front of each subject immediately before presentation and subjects were told prior to presentation whether it was a mixture of two or three different odors. The overall order of mixtures (from top to bottom) was implemented according to Table 2 (from left to right) and presented with an interval of at least 30 seconds between each mixture in order to prevent olfactory desensitization^61–63^. The investigator applied different Sniffin’ Sticks identification pens horizontally consecutively on a 6 × 6 cm standard piece of print paper (Canon, totally chlorine-free-TCF Copy Paper, 80 g/m²) over a length of 5 cm closely together (Fig. 6)^25,26^. In order to control for equal lengths and location of odor lines, examiners were also provided with a printed, graphical illustration (Fig. 6), additionally to the instruction to apply odor pens horizontally over a length of 5 cm closely together beginning at the upper end.

### The Sniffin’ Sticks test (TDI)

Olfactory performance was tested using the well-established Sniffin’ Sticks-test (Burghart Medical Technology, Wedel, Germany)^7,8^ according to the manufacturer’s instructions. The test consists of three subtests: Threshold (T), Discrimination (D), and Identification (I). Results of the Sniffin’ Sticks test were compared with normative data and all subjects included in this study (n = 88) were classified as normosmic (n = 51; TDI ≥ 30.75), hyposmic (n = 25; TDI less than 30.75 and higher than 16.0), or anosmic (n = 12; maximum TDI of 16.0) in comparison with normative data^27,28,31^.

### Statistical analysis

Results were analysed and graphically visualized using R Statistical Computing Software 3.4.4 (R Development Core Team, 2008; R Foundation for Statistical Computing, Vienna, Austria) and GraphPrism 8.2 (GraphPad Software, Inc., La Jolla, CA). For descriptive statistics, SSomix results of subjects with self-reported normal sense of smell (n = 66) from the first visit were separated into two previously described age groups as follows^27^: (i) those aged between 18-35 years (n = 54) and (ii) >35 (n = 12). Chance level responses were calculated based on the binomial probability distribution in Microsoft Excel 16.0.11929.20436 (Microsoft Office, Microsoft Corporation, Redmond, WA, USA).

Unpaired t-tests with Welch’s correction were used for group comparisons. Test-retest reliability was assessed using the intraclass correlation coefficient (ICC_3,1_) according to Shrout and Fleiss^29,45^. ICC levels were interpreted as follows: ICC ≥  0.9 was considered excellent reliability, 0.75  ≤ ICC  < 0.9 good, and 0.5 ≤ICC < 0.75 moderate^64^. Bland-Altman plots and 95% LOA were computed to confirm test-retest agreement^30^. Spearman’s rho was used for correlation analysis between SSomix and TDI scores and interpreted as follows: r(s) ≥  0.9 perfect correlation, 0.7  ≤ r(s)  < 0.9 strong, and 0.4 ≤ r(s) < 0.7 moderate^65,66^. Receiver operating characteristics (ROC) curves were plotted and area under the ROC curves (AUC) were calculated to determine diagnostic accuracy. AUC values were interpreted as follows: 0.9-1 = excellent accuracy, 0.8-0.9 = very good, 0.7-0.8 = good, and 0.6-0.7 = sufficient^67^. Youden’s Index was used to calculate optimal cut-off points^32^. The P-value was set at 0.05.

### Study limitations

The number of points to be expected for SSomix based on random response (corresponding to an anosmic patient) is 5 (equivalent to 17.2%; Fig. 5). Interestingly, anosmic patients in our study yielded SSomix scores slightly higher than 5, which could reflect residual olfactory function with no use in everyday life, or in accordance to a previously published study^68^, could be due to trigeminal traces included in some of the eleven odors used. However, due to the distribution of the SSomix scores obtained by anosmic patients in our study, it is not possible to determine whether a low SSomix score is due to olfactory dysfunction or malingering, which seems a general issue in chemosensory testing^69^.

Regarding the order of odor presentation, sequential positioning of some stimuli such as peppermint, might have introduced a potential bias relating to repeated presentation and inter-stimulus time^70,71^. However, the frequency of 30 seconds was chosen based on previous findings that odors can be reliably perceived based on this interstimulus-range^61–63^, hence repeated presentation of same odors might not have affected our results to a large extent.

As some odors, such as cinnamon, were used more often during the test protocol, inter-individual familiarity to odors might have also influenced our results. The potential bias of odor-familiarity in identification testing is well-known^72^, leading to a large number of culturally adapted and country-specific identification tests^33–42^. Therefore, further investigations are needed prior to cross-cultural testing and adaption, which may be performed easily, since different odors have already been assessed for familiarity as mentioned above.

Another limiting factor of this work is the “Odor-Lines-On-paper”^25,26^ method, which utilizes print paper and therefore bears the risk of an additional microbial contamination of the Sniffin’ Sticks resulting in an alteration of presented odors. However, a procedure using pens alone without the current method might also bear the risk of pen tips touching the nose or lips, since experience has shown that patients with severe olfactory dysfunction often (unintentionally) move their heads towards the Sniffin’ Sticks, expecting to smell more. Moreover, using pens simultaneously in a mixture test procedure also poses the uncertainty of tips touching each other, especially in regard to the possibility of self-administration. Therefore, future test instructions should place emphasis on this critical step and the description sequence in which to execute correctly the “Odor-Lines-On-Paper” method. Furthermore, the paper used for SSomix should be based on totally chlorine free copy paper with a weight of 80 g/m² in order to minimize the potential bias of paper type. Since Sniffin’ Sticks are based on a reusable procedure, microbial contamination can never be completely ruled out and further experimental studies are warranted on odor quality after repeated test cycles.

Despite the promising results, open questions remain including the direct comparison between SSomix and the 16-item Sniffin’ Sticks identification test as well as the additional diagnostic value of SSomix in patients with incongruent identification test results. However, to justify fully the use of SSomix as a stand-alone screening tool, additional work will be required in which both SSomix and the 16-item identification test need to be compared against the gold standard protocol (TDI). These limitations might be addressed by performing a prospective, diagnostic evaluation, recruiting patients with subjective smell loss but with identification test results within the normosmic range.

## Supplementary information

Supplementary information.

## Data Availability

The institutional ethics committee (Ethikkommission der Medizinischen Universität Wien - Borschkegasse 8b/E06, 1090 Vienna) is imposing legal and ethical restrictions on the present data. Requests for data will be administered by the corresponding author.
