# Supplementary material for: Odor Mixtures in Identification Testing Using Sniffin’ Sticks: The SSomix Test
Source: Sci Rep. 2020 May 18;10:8155. doi: 10.1038/s41598-020-65028-7 (PMC7234997; doi:10.1038/s41598-020-65028-7)
Supplement: Supplementary file 1 — Supplementary information. [file 41598_2020_65028_MOESM1_ESM.docx]

**Odor Mixtures in Identification Testing using Sniffin’ Sticks: The SSomix Test**

David Tianxiang Liu, Gerold Besser, Miriam Lang, Gunjan Sharma, Eleonore Pablik, Bertold Renner, Christian Albert Mueller

**Supplementary Table 1** Descriptive statistics and corresponding percentiles of SSomix results of the first visit obtained from subjects with self-reported normal sense of smell.

| **Age group A, 18-35 years, n = 54** |  |
| --- | --- |
| Mean | 18.6 |
| SD | 3.3 |
| Minimum | 11 |
| Maximum | 28 |
| Percentiles |  |
| 10 | 14 |
| 25 | 16 |
| 50 | 18.5 |
| 75 | 21 |
| 90 | 23.5 |
| **Age group B, > 35 years, n = 12** |  |
| Mean | 18 |
| SD | 3.9 |
| Minimum | 10 |
| Maximum | 24 |
| Percentiles |  |
| 10 | 10.9 |
| 25 | 14.8 |
| 50 | 19 |
| 75 | 20 |
| 90 | 23.4 |
